# NLRP3 Inflammasome Activation by Viroporins of Animal Viruses

**DOI:** 10.3390/v7072777

**Published:** 2015-06-24

**Authors:** Hui-Chen Guo, Ye Jin, Xiao-Yin Zhi, Dan Yan, Shi-Qi Sun

**Affiliations:** State Key Laboratory of Veterinary Etiological Biology, OIE/National Foot-and-Mouth Disease Reference Laboratory, Lanzhou Veterinary Research Institute, Chinese Academy of Agricultural Sciences, Lanzhou 730046, China; E-Mails: gguohuichen@caas.cn (H.-C.G.); jinye@caas.cn (Y.J.); zhixiaoyin333@163.com (X.-Y.Z.); danyan9005@163.com (D.Y.)

**Keywords:** viroporin, inflammasome, NLRP3, animal virus

## Abstract

Viroporins are a group of low-molecular-weight proteins containing about 50–120 amino acid residues, which are encoded by animal viruses. Viroporins are involved in several stages of the viral life cycle, including viral gene replication and assembly, as well as viral particle entry and release. Viroporins also play an important role in the regulation of antiviral innate immune responses, especially in inflammasome formation and activation, to ensure the completion of the viral life cycle. By reviewing the research progress made in recent years on the regulation of the NLRP3 inflammasome by viroporins of animal viruses, we aim to understand the importance of viroporins in viral infection and to provide a reference for further research and development of novel antiviral drugs.

## 1. Introduction

When infecting susceptible host cells, viruses undergo a series of processes to promote their attachment and entry into these host cells, to escape recognition of the host immune system, and to complete their subsequent life cycle. After viruses infect these host cells, the cell membrane permeability of the hosts increases; this increase in cell membrane permeability may facilitate viral invasion into these host cells. The cell membrane permeability of these hosts is also increased through the independent expression of specific viral proteins [1,2,3,4,5,6,7]. The viral proteins that can induce this increase in host cell membrane permeability are classified as viroporins [8,9]. These proteins are characterized by a low molecular weight and are composed of at least one hydrophobic region; viroporins are also usually present in the cell membrane as oligomers to form pores. With these structures, viroporins elicit similar biological effects on their host cells; for instance, these proteins increase the host cell membrane permeability and balance the disturbance among the corresponding intracellular ions (e.g., Na^+^, K^+^, Ca^2+^, Cl^−^, H^+^) [1,10,11,12]. As a result, inflammasomes are formed in host cells; apoptosis and autophagy are induced [13,14]; eventually, virus assembly and release are promoted.

Viroporins can form a channel after these proteins penetrate the cell membrane of host cells; this process is closely related to the structure of viroporins. Viroporins often contain one or two transmembrane (TM) domains; the difference between these TM domains results in the different membrane-crossing patterns of viroporins. Viroporins are also divided into two groups on the basis of the number of TM domains; each group is further divided into subgroups on the basis of the membrane-crossing characteristics of their N- and C-terminals and their corresponding topology. Viroporins can oligomerize very easily because of their structural properties. Once viroporins become an oligomer in the cell membrane, these proteins can form oligomer porins composed of viroporin proteins with different molecular numbers [15,16,17]. The formation of porins is necessary to promote virus invasion and release; viroporins are also implicated in virus release. In the invasion stage of some enveloped viruses, the modification of cell membrane permeability is primarily related to some glycoproteins [18,19]. However, the role of viroporins during the invasion of some non-enveloped viruses is not as important. Hence, whether other mechanisms or cellular proteins are involved in this process among different viruses should be investigated.

The increase in membrane permeability induced by viroporins can be demonstrated in two ways. In one of the mechanisms, hygromycin B or other small molecules and proteins with a molecular weight ranging from 800–1000 Da enter prokaryotic and eukaryotic cells after viroporins are expressed [20]. In the other mechanism, the balance of intracellular calcium ion concentration is disrupted; this effect is one of the most significant characteristics of viroporin-induced increase in membrane permeability. Changes in calcium ion concentration are mainly caused by either the entry of extracellular calcium into cells or the release of calcium from calcium storage sites, such as mitochondria, endoplasmic reticulum, and Golgi apparatus, into the cytoplasm. Ca^2+^ exchange between the endoplasmic reticulum and the mitochondria also plays an important role in the induction of apoptosis [13]; in the induction of apoptosis, excessively high Ca^2+^ concentrations can trigger the opening of mitochondrial membrane transition pores, induce the osmotic swelling of the mitochondria, and cause the rupture of the mitochondrial outer membrane [14,21]. As a result, cytochrome *c* and other pro-apoptotic factors are released and apoptosis occurs. Furthermore, the innate immune system of host cells possibly responds to the disturbance of the balance of ion concentration and stimulates immune activation signals to counteract viral infection [22]. Subsequently, it will be mainly described that viroporins involve in inflammasome activation and induce the innate immune response in the next chapter.

## 2. Inflammasomes

**Figure 1:**
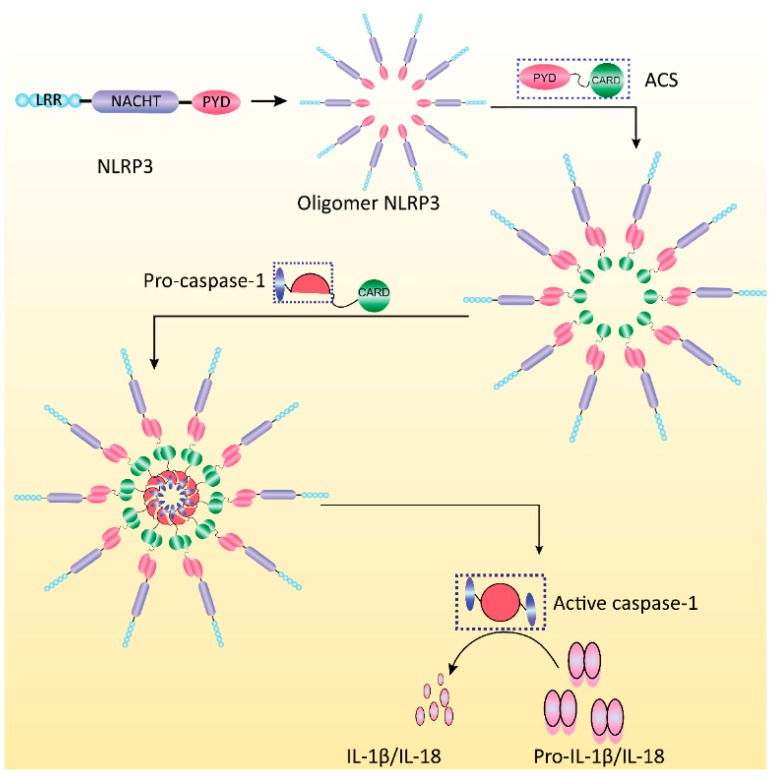
Illustration of the formation and activation of the NLRP3 inflammasome. The NLRP3 monomer can be activated by its ligands and oligomerized to form a defined oligomer. The recruitment of ASC (apoptosis-associated speck-like protein containing a caspase recruitment domain) is the linkage of NLRP3 with the recruited pro-caspase-1 through PYD and CARD domains. Pro-caspase-1 autoactivates to become an active caspase-1, which then cleaves the pro-inflammatory cytokines pro-IL-1β and pro-IL-18 to produce active cytokines.

To respond to infection and changes in the metabolic and cellular stress caused by intracellular damage, host cells recognize invasive microorganisms and viruses through the expression of germline-encoded pattern recognition receptors (PRRs) [23,24,25].These invasive components are referred to as pathogen-associated molecular patterns (PAMPs) [26]. PRRs mainly include Toll-like receptors (TLRs) [27], retinoic acid inducible gene-I (RIG-I)-like receptors [28], nucleotide-binding oligomerization domain (NOD)-like receptors (NLRs), and are absent in melanoma 2 (AIM2)-like receptors(ALRs) [29]. As a type of PRR, NLRs can recognize PAMPs and damage-associated molecular patterns (DAMPs) [30]. Based on their different N-terminal domains, NLRs can be divided into five subfamilies: NLRs with acidic activation domains, NLRPs with baculovirus IAP repeat domains, NLRs with caspase recruitment domains (CARDs), NLRs with PYD domains (N terminal PYRIN domain, NLRPs/NALPs), and NLR family members that do not show strong homology to the N-terminal domains of any other NLR subfamily member (NLRXs) [31,32]. NLRs play an important role in basic cellular life events, such as apoptosis, autophagy, and development. Additionally, NLRs are an important component of the so-called “inflammasomes” inside the cell [33] (Figure 1).

Inflammasomes are a group of protein complexes that exist in the cytoplasm or in the nucleus. Once activated, inflammasomes can recruit caspases to trigger the secretion of the inflammatory cytokines IL-1β/IL-18, which subsequently induce inflammatory responses [34]. Inflammasomes are composed of three major components: inflammatory signal recognition receptors, ASC adaptor protein, and caspase-1 precursor. Inflammasomes perform two different functions that involve caspase-1: (1) inflammasomes cleave and activate inflammatory cytokines; (2) inflammasomes also induce pyroptosis. Pyroptosis is a caspase-1-mediated special programmed cell death, which is morphologically different from traditional apoptosis. Inflammasomes recognize various ligands, including bacteria, viruses, fatty acids, and crystal structures. Inflammasomes are also an important component of the innate immune system, and these substances are the primary immune barrier that defends host cells from exogenous pathogen infection and prevents stimulation by harmful endogenous factors. Inflammasomes can be activated when viruses infect target cells; as a result, local inflammatory responses and subsequent immune responses are induced [35]. However, viruses use numerous strategies to evade recognition by inflammasomes and to replicate inside host cells.

## 3. NLRP3 Inflammasome

NLRP3 is a unique molecule and the most clearly studied among the members of the NLR family. NLRP3 often exists in an inactive form in the cytoplasm. This molecule is activated through the following mechanism. The C-terminal leucine-rich repeats (LRR) of NLRP3 become folded; this process allows NLRP3 to approach the NACHT/NOD domain, which blocks the activation site; as a result, NLRP3 is maintained in an inactive state. PAMP and other pathogen signals directly or indirectly bind to the LRR in the signal recognition region. The binding triggers a conformational change in NLRP3 to expose the hidden NACHT/NOD domain, which further induces the NLRP3 molecule to oligomerize and form hexamer or heptamer structures. The PYD effector domain located at the N-terminus of NLRP3 becomes exposed; the PYD domain recruits the nearby ASC adaptor protein and then allows the ASC adaptor protein to bind to the NLRP3 protein through a PYD–PYD interaction [36]. The ASC molecule also oligomerizes to form ASC dimers because of the interaction with an upstream signal recognition receptor; multiple ASC dimers subsequently assemble to form a large protein polymer. The large ASC polymer recruits pro-caspase-1, which exists as a pro-enzyme in the cytoplasm, through CARD-CARD interactions. Afterward, the ASC polymer induces the self-cleavage and activation of the recruited pro-caspase-1 to form an active caspase-1 hetero-tetramer. The activated caspase-1 subsequently cleaves pro-IL-1β and pro-IL-18 [37], which also exist as pro-enzymes in the cytosol. The activated IL-1β and IL-18 are then secreted to the extracellular space through a non-traditional secretory pathway to participate in downstream inflammation [38] (Figure 1).

The activation of NLRP3 may involve three types of signals. The first type of signal is produced through the activation of PRRs, such as TLRs and NLRs, and cytokine receptors, such as IL-1 and TNF receptors, which subsequently stimulate the transcriptional expression of pro-IL-1β, pro-IL-18, and NLRP3 [32,39,40]. The second type of signal is induced by a series of PAMPs and DAMPs, such as the cell membrane damage caused by bacterial toxins, ATP, potassium efflux, and pore formation; the second type of signal is also stimulated by lysozyme instabilities induced by silicon dioxide, alum, β amyloid protein, uric acid crystals, and other granular activators; as a result, cathepsin B is released into the cytoplasm, thereby activating NLRP3. The third type of signal is caused by reactive oxygen species (ROS) signaling [41,42]; in this process, the NLRP3 inflammasome is activated by ROS and calcium influx is mediated by the M2 protein channel potential [43].

**Figure 2:**
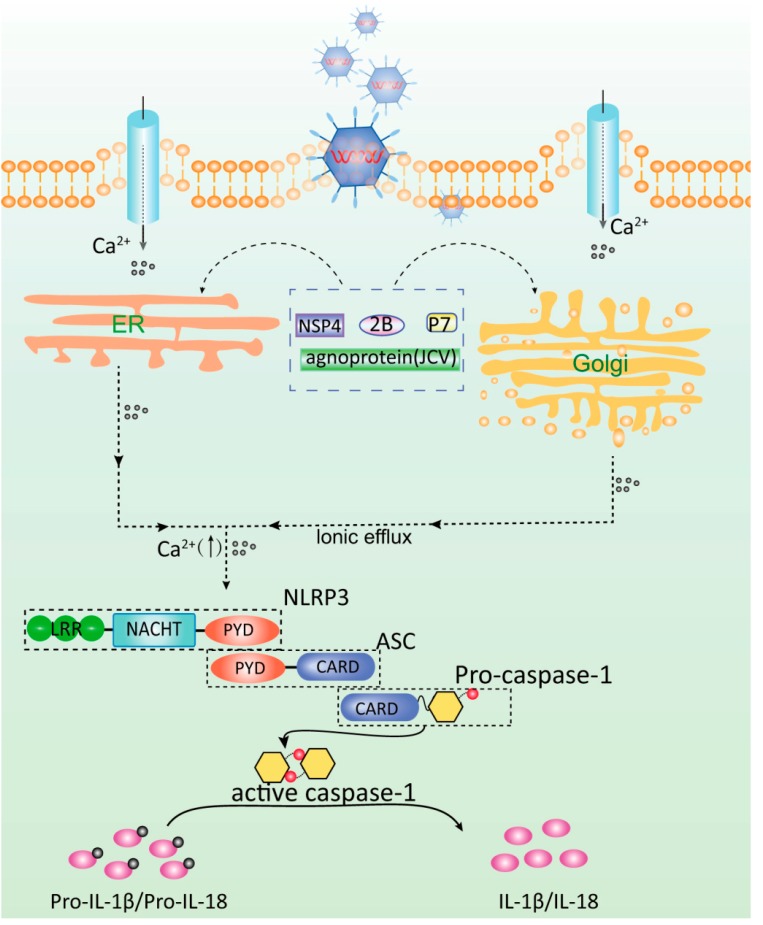
Dysregulation of ions leads to the activation of the NLRP3 inflammasome, as induced by viroporin expression. Viroporin generally permeabilizes intracellular compartments, such as the Golgi and the *trans*-Golgi network, and the plasma membrane. Viroporin induces the imbalance of ions, such as Ca^2+^ (e.g., NSP4 [10,47], p7 [48,49], 2B [5,50], Agnoprotein [4,51]) by several ways, in which free cytosolic Ca^2+^ is increased through (**1**) the entry of extracellular Ca^2+^ across the plasma membrane via the modulation of Ca^2+^ pumps or channels on the plasma membrane or (**2**) the release of Ca^2+^ from the endoplasmic reticulum, the Golgi complex, lysosomes, or mitochondria. However, only Rhinovirus 2B protein [52], EMCV 2B protein [50] and HCV p7 protein [53] regulated Ca^2+^ concentration were recently reported to activate the NLRP3 inflammation by regulating Ca^2+^ concentration.

The flow of Ca^2+^ also induces the activation of the NLRP3 inflammasome [44,45]. Extracellular calcium concentration is increased by G protein-coupled calcium-sensing receptors (CaSRs) or G protein-coupled receptor family C group 6 member A; the extracellular calcium concentration is also increased when phospholipase C hydrolyzes phosphatidylinositol 4,5-bisphosphate to produce diacyl glycerol and inositol trisphosphate (InsP3, IP3); these substances then activate the NLRP3 inflammasome. InsP3 also induces the release of calcium from the endoplasmic reticulum via the InsP3 receptor [46]; as a result, intracellular calcium concentration is increased. InsP3 further promotes inflammasome assembly and induces IL-1β production. The release of calcium from the endoplasmic reticulum induces a store-operated Ca^2+^ entry-dependent calcium influx from the extracellular matrix; this process is also necessary to activate the NLRP3 inflammasome. Furthermore, the activated CaSRs induce a decrease in intracellular cAMP. In addition, activated CaSRs induce a decline of intracellular cAMP, thereby activating the NLRP3 inflammasome in a dependent manner.The IL-1β release and calcium influx induced by charged liposomes are also dependent on ROS. The deficiency or inhibition of transient receptor potential melastatin 2, a non-selective cationic channel, blocks the calcium influx- and liposome-induced activation of the NLRP3 inflammasome [32] (Figure 2). Therefore, calcium is implicated in inflammasome activation.

## 4. NLRP3 Inflammasome Activation by Viroporins

After infecting host cells, viruses use or interfere with host intracellular ion transport and ion signal transduction systems to successfully replicate and proliferate inside the host cells. Viroporins increase the permeability of the cellular, endoplasmic, and mitochondrial membranes; this increase causes an efflux of ion from these organelles to the cytoplasm, resulting in an increased ion concentration in the cytoplasm. The increased ion concentration subsequently activates or accelerates ion-dependent enzyme reactions and ion-sensitive transcriptional factors in the cytosol; viral replication and transcription are then promoted. Thus, viruses can complete their replication cycle in the host cells. Furthermore, calcium is implicated in inflammasome activation; as such, viroporins may also play an important biological role in ion-induced activation of the NLRP3 inflammasome [21,22,38].

Although multiple virus-encoded proteins exhibit viroporin properties, the involvement of these viroporins in the activation of the NLRP3 inflammasome is rarely reported. These proteins mainly include Respiratory syncytial virus (RSV) small hydrophobic (SH) protein, Influenza virus M2 protein, Rhinovirus 2B protein, Encephalomyocarditis virus (EMCV) 2B protein and Hepatitis C virus (HCV) p7 protein.

RSV can cause cells to produce IL-1β. *In vitro*, RSV can trigger the activation of the NLRP3/ASC inflammasome via the following mechanisms: after host cells are infected, TLR2/myeloid differentiation primary response 88 (MyD88)/nuclear factor κB (NF-κB), which is the first signal, is activated; afterward, the NLRP3/ASC inflammasome, together with the second signal from ROS and a potassium current, is activated. Both signals induce caspase-1 activation and subsequent IL-1β secretion [54]. The RSV SH protein is a viroporin that can enhance membrane permeability and promote the entry of ions and small molecules into host cells through the membrane pores formed by by its oligomers. An SH-deficient RSV mutant cannot induce the activation of the NLRP3/ASC inflammasome and the subsequent secretion of IL-1β; as such, the RSV SH is necessary to activate the NLRP3 inflammasome. RSV-infected cells can be treated with drugs that inhibit viroporin activity; the treatment also suppresses inflammasome activation. This result suggests that SH viroporin or lipid rafts are involved in inflammasome activation. After RSV infects host cells, the RSV SH protein accumulates in the lipid rafts of the Golgi apparatus, thereby forming a positive ion-permeable specific pore. These positive ion fluxes induce the transport of NLRP3 from the cytosol to the Golgi apparatus and promote the NLRP3 activation. However, these studies have confirmed the involvement of SH viroporin in inflammasome activation on the basis of this phenomenon. The detailed mechanism of the inflammasome activation induced by RSV SH should be further investigated [55].

Although the RNA of the influenza virus can stimulate TLR7 signaling and pro-IL-1β transcription, the M2 protein of the influenza virus can act as a second signal to stimulate the activation of the NLRP3 inflammasome [56]. The M2 protein is a viroporin involved in viral genome replication and assembly, as well as in viral particle entry into and release from host cells. Furthermore, the M2 protein allows the entry of protons into virions; thus, virus uncoating in endosomes is promoted. The M2 protein not only exhibits viroporin properties but also regulates the pH balance between the lumen of the *trans*-Golgi complex and the cytosol; as a result, the acidity of intracellular vesicles and the acidity inside the organelles are reduced. Wild-type influenza virus can also stimulate host cells to produce IL-1β and IL-18 after these host cells are infected, and a recombinant retrovirus expressing the M2 protein can activate the NLRP3 inflammasome in LPS-stimulated cells. The histidine 37 residue in the transmembrane domain of the M2 protein is critical for the proton selectivity of the M2 protein. The transduction of an LPS- or poly (I:C)-stimulated cell line with a retrovirus harboring a mutation at this position shows that the levels of IL-1β induced by this mutant recombinant virus are higher than those of the wild-type M2 recombinant retrovirus. Moreover, the cells infected by the wild-type influenza strain are treated with monesin (a Na^+^-H^+^ anti porter in the *trans*-Golgi network); in these cells, the secretion of IL-1β and the accumulation of the M2 protein in the Golgi apparatus are significantly enhanced. The Golgi apparatus-disrupting drug Brefeldin A can block IL-1β release and induce the M2 protein to localize in the endoplasmic reticulum. Therefore, the cellular localization and the biological activity of the M2 viroporin are implicated in NLRP3 inflammasome activation.

EMCV viroporin 2B also plays an important role in NLRP3 inflammasome activation. The expression of the 2B protein can induce IL-1β release, 2B can co-localize with NLRP3 in the perinuclear space; thus, the NLRP3 protein can undergo cellular translocation. The ion channel activity of the 2B protein decreases the calcium concentration in the endoplasmic reticulum and the Golgi apparatus; this decrease significantly affects IL-1β secretion. In addition, thapsigargin and the Ca^2+^ ionophore ionomycin significantly increase the calcium concentration in the cytosol and further induce IL-1β release into EMCV-infected cells. By contrast, the calcium chelator BAPTA-AM inhibits IL-1β release induced by EMCV infection or 2B protein expression [50].

Human rhinovirus (HRV) infection or 2B protein expression can also induce NLRP3-dependent caspase-1 cleavage and IL-1β production. In respiratory tract cells, which are either infected by HRV or transfected with the 2B protein alone, NLRP3 binds to NLRC5 and ASC, which is co-localized with NLRC5 and the 2B protein in the Golgi apparatus [52]. Cells were treated with Brefeldin A, in which 2B protein or a 2B protein containing ER-targeting signal sequence over-expressed, demonstrating the significant inhibition of IL-2β release. HRV-infected cells are also treated with BAPTA-AM or verapamil; this treatment also inhibits IL-1β release and caspase-1 activation.

Hepatoma Huh7.5 cells or THP-1 macrophages infected with hepatitis C virus (HCV) induces IL-1β production and NLRP3 inflammasome activation [53,57]. The ROS inhibitor diphenyleneiodonium and the potassium channel inhibitor glibenclamide block HCV-induced IL-1β production. The genomic RNA of HCV and p7 protein also triggers NLRP3 inflammasome activation; this genomic RNA also induces the oligomerization of ASC and the maturation of caspase-1 in THP-1 cells via NLRP3 [53]. The production of IL-1β can be blocked by the ROS inhibitor in a dose-dependent manner. Therefore, HCV induces NLRP3 inflammasome activation via the genomic RNA of HCV and the ROS model [58].

In summary, the viroporin activity of viruses can disrupt the balance of intracellular ion concentration. Responses to this imbalance activate the NLRP3 inflammasome. However, ion channel protein blockers affect this activation process. Hence, viroporins can represent a novel group of molecules that activate the NLRP3 inflammasome. Specific viroporin blockers or ion chelators can be used as effective antiviral drugs.

## 5. Concluding Remarks

Although studies have been extensively conducted to investigate the biological function of viroporins, studies on the involvement of these proteins in NLRP3 inflammasome activation have been rarely performed. The detailed molecular mechanism of NLRP3 inflammasome activation by viroporin remains unclear. Studies have also described viruses encoding viroporins; nevertheless, few reports have demonstrated the relationship between and the molecular mechanism of viroporins and the NLRP3 inflammasome. Therefore, further studies are warranted to determine whether viroporins are involved in inflammasome activation. Specific cellular proteins, as well as viral proteins (such as viroporins), participate in virus-induced NLRP3 activation. However, the detailed mechanisms should be further investigated. The molecular mechanism of inflammasome activation by viroporin should also be elucidated. With this development, viroporins can be used in antiviral applications.
